# Angiographic prevalence of occlusion myocardial infarction and the limited value of serial troponin in patients with non–ST-segment elevation acute coronary syndrome

**DOI:** 10.3389/fcvm.2026.1903711

**Published:** 2026-10-05

**Authors:** Sajid Kadir, Sandeep Sasidharan, Sandipan Shringi, Mahati Dasari, Pramukh Arun Kumar

**Affiliations:** 1 Pepin Heart Institute, AdventHealth Tampa, Tampa, FL, United States; 2 Department of Internal Medicine, University of Florida College of Medicine, Jacksonville, FL, United States; 3 Department of Internal Medicine, Warren Alpert Medical School of Brown University, Providence, RI, United States; 4 Department of Cardiovascular Medicine, University of Massachusetts Chan Medical School – Baystate, Springfield, MA, United States; 5 Calhoun Cardiology Center, University of Connecticut Health, Farmington, CT, United States

**Keywords:** coronary angiography, NOMI, non–ST-elevation acute coronary syndrome, occlusion myocardial infarction, OMI, troponin

## Abstract

**Background:**

An estimated 25%–34% of patients with non–ST-segment elevation acute coronary syndrome (NSTE-ACS) are diagnosed with an occluded culprit artery during catheterization. Whether early serial troponin kinetics discriminate angiographically defined occlusion myocardial infarction (OMI) in catheterized patients with NSTE-ACS remains uncertain.

**Methods:**

We conducted a single-center retrospective cohort study of 481 patients with NSTE-ACS who underwent coronary catheterization between 2017 and 2023. OMI was defined by a purely angiographic criterion: >90% culprit-vessel stenosis with Thrombolysis in Myocardial Infarction flow grade 0, 1, or 2. Seven contemporary sensitivity troponin I metrics (absolute values at 0, 3, and 6 h; deltas; and ratios) were evaluated by receiver operating characteristic analysis. Operating characteristics are reported with 95% confidence intervals (CIs) and likelihood ratios.

**Results:**

Ninety-eight patients (20.4%) met the angiographic definition of OMI. These patients were younger (median, 65.5 vs. 70.0 years; *p* = 0.025), had fewer comorbidities, and more often presented with chest pain (92.9% vs. 82.8%; *p* = 0.013). The left circumflex artery was the most common culprit in OMI (36.7%). Twenty-nine patients with OMI (29.6%) had a presentation ECG classified as normal (no ST–T change or bundle branch block. No troponin metric reliably discriminated OMI from non-occlusion MI. The prespecified primary predictor, the 0–6-h change in troponin concentration, had an area under the curve (AUC) of 0.623 (95% CI, 0.544–0.703) and the best single metric, the 6-h value, of 0.654 (0.580–0.727); all seven unadjusted troponin-metric AUCs were under 0.66. At its Youden cutoff (≥0.379 ng/mL), the 6-h troponin had a sensitivity of 69.4%, a specificity of 57.7%, a positive predictive value of 27.2%, and a negative predictive value of 89.3%. The highest positive predictive value of any metric was 41.3%, the first troponin (at 0 h) identified fewer than 40% of patients with OMI at its optimal threshold, and likelihood ratios produced only limited shifts in post-test probability.

**Conclusion:**

Among analyzable patients with NSTE-ACS undergoing catheterization, approximately one in five met the study-defined angiographic criteria for OMI. Early serial troponin kinetics showed only modest discrimination, and upper confidence bounds fell below levels expected of a standalone triage biomarker. Complementary strategies for identifying these patients, including expert ECG interpretation, warrant further study.

## Introduction

Acute coronary syndrome (ACS) is a leading cause of cardiovascular morbidity and mortality worldwide (1, 2). The current clinical framework classifies ACS into ST-segment elevation myocardial infarction (STEMI) and non–ST-segment elevation ACS (NSTE-ACS), with the latter further divided into two types: non–ST-segment elevation myocardial infarction (NSTEMI) and unstable angina. This dichotomy rests on the assumption that ST elevation on the electrocardiogram (ECG) serves as a reliable indicator of acute coronary occlusion, thereby identifying patients who require emergent reperfusion therapy (3, 4).

There is growing evidence that this framework misses an important group of patients. Meta-analysis data from over 40,000 patients with NSTEMI demonstrate that about 25% of patients with NSTEMI have a totally occluded culprit artery at the time of coronary angiography, with this proportion increasing to 34% when near-total occlusion is included (5, 6). These patients do not undergo emergent reperfusion; however, their mortality rate doubles compared to patients with NSTEMI without occlusion, despite their younger age and fewer comorbidities (5, 7). Conversely, the sensitivity of conventional STEMI criteria for detecting acute coronary occlusion is estimated to be only 35%–44%, meaning that a large share of patients with an occlusive pathology are triaged toward a non-urgent invasive strategy (8, 9).

These observations have given rise to the occlusion myocardial infarction (OMI) paradigm, which redefines the classification of acute MI based on the presence or absence of acute coronary occlusion rather than on ST-segment millimeter thresholds (10, 11). Under the OMI framework, a subset of patients classified as having NSTEMI in fact have the same underlying pathology as STEMI, an acutely occluded coronary artery, but lack the characteristic ECG findings that would trigger emergent catheterization. As a result, these patients may experience clinically meaningful delays in revascularization, with potential consequences for myocardial salvage and long-term outcomes (7).

Most work on OMI so far has focused on ECG interpretation, particularly the recognition of occlusion patterns that do not meet classic STEMI criteria (11). Few studies have examined angiographically confirmed OMI in NSTEMI cohorts or investigated readily available clinical markers that might identify these patients before they undergo angiography. Serial cardiac troponin measurement is a cornerstone of ACS evaluation. Its role in distinguishing occluded from non-occluded coronary pathology has not been well characterized.

In this study, we sought to determine the angiographic prevalence of OMI among patients presenting with NSTE-ACS, evaluate the discriminative performance of early serial troponin kinetics, and compare baseline clinical features between patients with OMI and those with non-occlusion MI (NOMI). We evaluated troponin as a candidate risk-enrichment marker among patients already diagnosed with NSTE-ACS, not as a standalone rule-in or rule-out test. Then, we compared all-cause mortality between OMI and NOMI groups as an exploratory analysis.

## Materials and methods

We conducted a single-center retrospective cohort study at Saint Vincent Hospital, Worcester, Massachusetts. Saint Vincent Hospital is an urban community teaching hospital serving central Massachusetts, with on-site cardiac catheterization laboratories, facilities for primary percutaneous coronary intervention (PCI) and cardiothoracic surgery, and accredited cardiology and interventional cardiology fellowship programs. The study period extended from 1 January 2017 to 31 December 2023. All adult patients (≥18 years) who underwent coronary catheterization with a clinical indication of NSTE-ACS were identified from the institutional cardiac catheterization registry. Patients catheterized for indications other than NSTE-ACS, those without an identifiable acute culprit lesion, and those adjudicated as having type 2 myocardial infarction (12) were excluded. Classification required verifiable culprit-vessel flow. Patients who met the >90% stenosis criterion were classified based on their recorded Thrombolysis in Myocardial Infarction (TIMI) flow grade. Patients without a recorded TIMI flow grade were retained only if the catheterization report documented 100% occlusion of the culprit vessel (treated as equivalent to absent flow), whereas those with neither a recorded TIMI grade nor documented 100% occlusion were excluded because the angiographic OMI status could not be verified. This study was approved by the Institutional Review Board, and individual informed consent was waived given the retrospective observational design of the study. This study is reported in accordance with the STROBE (Strengthening the Reporting of Observational Studies in Epidemiology) guidelines (13).

This study was conducted before the implementation of an institutional OMI-directed triage protocol. Catheterization decisions therefore reflected conventional STEMI/NSTEMI-based pathways rather than a standardized OMI strategy. Patients were therefore managed under the prevailing guideline framework, in which patients with NSTEMI are recommended an early invasive strategy unless they are hemodynamically unstable or have refractory symptoms (3, 4).

### Data collection

Clinical data were extracted from the electronic medical record by trained investigators using a standardized data collection form. Baseline variables included age, sex, race, and the following comorbid conditions: hypertension, hyperlipidemia, diabetes mellitus, renal dysfunction, peripheral vascular disease, chronic lung disease, heart failure, and a history of cancer. Cardiac history variables included prior myocardial infarction, prior PCI, and prior coronary artery bypass grafting (CABG). Symptoms at presentation (chest pain, active chest pain, dyspnea, nausea, or vomiting) and the history of tobacco use (never, former, or current) were recorded. Renal dysfunction, heart failure, and chronic lung disease were recorded as binary comorbidity variables during the original standardized chart abstraction; the granular operational definitions used in the original abstraction form were not retained in the analytic dataset and cannot now be reconstructed. Chest pain at presentation denoted chest pain as a presenting symptom of the index illness. Active chest pain was recorded as a separate binary variable during abstraction; similarly, the precise operational wording used on the original form was not retained. Active pain status was available for 80 patients with OMI and 342 patients with NOMI. Abstractors recorded raw clinical and angiographic fields and were not blinded to troponin values; OMI status was not assigned by abstractors but was computed afterward from the abstracted stenosis and TIMI flow fields. The proportion of records abstracted in duplicate was not recorded.

### Troponin measurement

Serial cardiac troponin I (cTnI) levels were measured using the Abbott ARCHITECT STAT Troponin-I assay (Abbott Diagnostics, Abbott Park, IL, USA), a contemporary sensitivity assay with a limit of detection (LOD) of 0.01 ng/mL and a 99th-percentile upper reference limit (URL) of 0.03 ng/mL. Troponin was drawn according to the institutional protocol at presentation and at approximately 3 and 6 h thereafter; these are referred to throughout this study as the first, second, and third troponin values. The first value was the troponin drawn at initial presentation and served as the time 0 reference against which subsequent intervals were defined. Time 0 is therefore an anchor of hospital presentation rather than of symptom onset, and the time from symptom onset was not recorded. During the original abstraction, draw times were checked against clinical timestamps solely to determine whether measurements fell within the prespecified sampling windows. The exact draw timestamps and draw-to-angiography or draw-to-PCI intervals were not retained in the analytical dataset. A second or third value was recorded only if a draw fell within ±30 min of the protocol-specified 3- or 6-h time point, respectively; draws outside these windows were not abstracted, thereby ensuring comparable intercollection intervals across patients. A later value is missing whenever no draw fell within the window (e.g., in patients catheterized before the 6-h window), rather than because sampling did not occur clinically. Analyses were performed using metric-specific available-case populations.

Laboratory results fell into three categories for coding purposes. Results reported as undetectable (i.e., below the analytic measurement range; recorded as zero in the retained analytic dataset) were coded at the LOD (0.01 ng/mL). Results reported as “<0.030,” indicating a detectable signal above the LOD but below the reportable range, were coded at the URL (0.03 ng/mL). All other results were entered at their reported numeric value. These coding decisions were applied uniformly regardless of the OMI status.

The troponin trajectory was characterized using two derived metrics: the delta troponin (later value minus first value) and the troponin ratio (later value divided by first value). Delta troponin was calculated over two intervals: 0 → 3 h and 0 → 6 h. The ratio was calculated for both the 0 → 3-h (second ÷ first) and 0 → 6-h (third ÷ first) intervals.

For the categorical conversion analysis, an initially undetectable troponin was defined as a first value coded at the LOD (0.01 ng/mL), that is, a result reported by the laboratory as undetectable or zero. Patients with a first value of “<0.030” (coded at 0.03 ng/mL) were not classified as undetectable because they had a measurable signal above the LOD, even though the result fell below the clinical positivity threshold. Conversion was defined as any subsequent value ≥0.03 ng/mL on the second or third draw.

Peak troponin concentration during the full hospitalization was not systematically captured and cannot currently be retrieved. The values analyzed represent only the early serial measurements obtained during the initial evaluation period and should not be interpreted as peak values.

### Electrocardiographic and echocardiographic data

The initial 12-lead ECG at presentation was classified into four mutually exclusive categories: normal, ST–T-wave changes (ST depression and/or T-wave inversion), left bundle branch block (LBBB), or right bundle branch block (RBBB). Adjudication was performed against the source ECG in case of any data-entry discrepancies. Patients with a recorded ECG but no positive finding in any of the three abnormal categories were classified as normal. Transthoracic echocardiographic findings were recorded as normal or reduced left ventricular systolic function, defined as an ejection fraction (EF) <50%.

### Coronary angiography and definition of occlusion myocardial infarction

Coronary angiographic findings were abstracted from the cardiac catheterization report. The severity of stenosis was recorded as a percentage for each major epicardial vessel: left main, left anterior descending artery (LAD), right coronary artery (RCA), ramus intermedius, and obtuse marginal/left circumflex artery (OM/LCx). The TIMI flow grade was recorded for the culprit vessel. The culprit lesion was identified in the catheterization report as the vessel designated by the interventional cardiologist as responsible for the acute presentation. Lesions described as chronic total occlusions, prior known occlusions, bypassed chronic native vessel occlusions, or non-culprit chronic disease were not considered culprit lesions unless the report explicitly identified them as the cause of the acute event. Single-vessel vs. multivessel coronary artery disease was recorded. For patients with NOMI, the culprit vessel was recorded as designated by the interventional cardiologist in the catheterization report; in patients with multivessel disease and no single clearly acute lesion, the vessel treated with PCI was recorded as the culprit; and if no intervention was performed, no culprit vessel was assigned. Troponin concentrations were generated by the clinical laboratory via an automated analyzer without reference to angiographic findings, so the index test was measured while being blinded to the reference standard. The reference standard was abstracted from the clinical catheterization report of the treating interventional cardiologist who was not blinded to clinical information, including troponin results available at the time of the procedure. No independent blinded angiographic adjudication was performed.

OMI was defined by a purely angiographic criterion applied to the identified culprit lesion: >90% stenosis and a TIMI flow grade of 0, 1, or 2. This definition was chosen for two reasons. First, requiring both high-grade stenosis and reduced flow ensures identification of hemodynamically significant epicardial occlusion, excluding slow flow due to microvascular dysfunction, distal embolization, or no-reflow without a severe proximal culprit. Second, because the primary aim of this study was to identify troponin-based predictors of OMI, a troponin-free outcome definition was used to avoid incorporation bias. This purely angiographic approach is more conservative than the composite definition (TIMI 0–2, or TIMI 3 with a highly elevated peak troponin concentration) used in prior OMI paradigm studies (10, 11) and may underestimate true OMI prevalence by excluding patients with spontaneous reperfusion to TIMI 3 flow prior to angiography (14, 15). We accepted this tradeoff to keep troponin out of the outcome definition. Consistent with established OMI definitions (10, 11), the angiographic criterion used a TIMI 0–2 threshold. We performed two *post hoc* sensitivity analyses of the occlusion boundary. The first analysis restricted the OMI group to TIMI 0–1 (*n* = 72: 57 with TIMI 0, 10 with TIMI 1, and 5 with documented 100% occlusion and TIMI flow not formally recorded), excluding the 26 patients with TIMI 2 flow. The second analysis reassigned these 26 patients to the non-occlusion group—the more conservative treatment of the boundary.

Patients who did not meet the aforementioned criteria were classified as having NOMI. The NOMI group was therefore heterogeneous, encompassing patients with NSTE-ACS and an identified culprit lesion that did not meet the angiographic OMI definition, including non-occlusive culprit lesions and high-grade lesions with preserved TIMI 3 flow. No further subclassification of NOMI was performed.

### Catheterization timing

Time to catheterization was calculated from presentation, operationalized as the timestamp of the first troponin draw, to catheterization. Patients who underwent catheterization more than 24 h after presentation were classified as having delayed catheterization; this threshold is consistent with the upper bound of the guideline-recommended early invasive strategy for high-risk NSTE-ACS (3, 4). Catheterization timing was anchored to the presentation troponin draw during the original abstraction and was available for the full cohort; the corresponding troponin concentration was unavailable for two patients with NOMI.

### Outcome measures

The primary outcomes were (1) the angiographic prevalence of OMI among patients with NSTE-ACS undergoing catheterization and (2) the discriminative performance of troponin-based metrics for the prediction of OMI, with the change in troponin from 0 to 6 h (delta 0 → 6 h) designated *a priori* as the primary predictor of interest. The intended clinical role evaluated was risk enrichment for OMI among patients with NSTE-ACS, to inform prioritization for urgent invasive evaluation, rather than as a standalone rule-in or rule-out test.

Secondary outcomes included all-cause mortality reported at time horizons for which outcome ascertainment reached at least 85% of the cohort. Time points with lower ascertainment were not analyzed. Cause-specific (cardiovascular) mortality was not reported because retrospective cause-of-death attribution was unreliable in this dataset. All mortality analyses are designated as exploratory; the cohort was not powered for mortality endpoints. No *a priori* sample size calculation was performed; the study size was determined based on the number of patients meeting the eligibility and data availability criteria during the study period.

### Statistical analysis

Continuous variables are expressed as median (interquartile range) and compared using the Mann–Whitney *U*-test. Categorical variables are expressed as frequency (percentage) and compared using the Pearson *χ*^2^ test; for 2 × 2 comparisons, the Fisher exact test was used when expected cell counts were small and for all mortality comparisons. All tests were two-sided, with *p* < 0.05 considered statistically significant. No adjustment for multiple comparisons was applied; baseline comparisons are therefore interpreted as a coherent clinical profile rather than as individually confirmed associations.

The troponin analysis comprised three components: comparison of absolute values at each draw, comparison of trajectory metrics (delta and ratio), and comparison of conversion rates among patients with an initially undetectable troponin concentration. The change in troponin concentration from 0 to 6 h (delta 0 → 6 h) was the prespecified primary discrimination analysis; all other metrics were secondary. Discriminative performance was assessed by the area under the receiver operating characteristic curve (AUC). The 95% confidence interval (CI) for each AUC and the test of each AUC against the no-discrimination value of 0.50 were computed using the DeLong method. Cutoffs selected by the Youden index (maximizing sensitivity + specificity − 1), along with their corresponding sensitivity, specificity, positive predictive value, and negative predictive value, are reported as apparent in-sample operating characteristics; because each cutoff was both derived and evaluated in the same sample, these are optimistically biased best-case estimates and are hypothesis-generating only, requiring external validation before clinical application.

Each metric was analyzed in the subset of patients with the relevant draws; these metric-specific diagnostic cohorts are reported with each estimate. Wilson score 95% confidence intervals were computed for sensitivity, specificity, and predictive values, and log-method intervals were computed for likelihood ratios. AUCs were compared using paired DeLong tests on shared samples. Because conditions such as renal dysfunction and heart failure can elevate troponin concentration independently of acute coronary occlusion (12, 16) and because there are age differences between groups, exploratory logistic models related OMI to the log_10_-transformed second and third absolute troponin concentrations, adjusted for age and serum creatinine; the delta metrics can be negative or zero, so a log-scale model is undefined for them, and the prespecified primary predictor was therefore additionally modeled on its original scale, with winsorization at the 1st and 99th percentiles as a robustness check. Additional sensitivity analyses comprised the two TIMI reclassifications described above and restriction to patients catheterized more than 24 h after the presentation, in whom all serial samples were obtained before catheterization. Within the OMI group, troponin concentration was compared across the three major culprit territories using the Kruskal–Wallis test. For interpretation, we regarded an AUC of approximately 0.80 as the minimum for an actionable triage biomarker, consistent with conventional descriptors under which 0.7–0.8 represents acceptable and 0.8–0.9 represents excellent discrimination (17). This benchmark was adopted during revision and is interpretive rather than prespecified.

Analyses were performed in Python (version 3.12) using SciPy (version 1.14), scikit-learn (version 1.5), and statsmodels (version 0.14).

## Results

### Study population

Between 1 January 2017 and 31 December 2023, 1,260 coronary catheterizations were performed at the study institution. After excluding 539 patients catheterized for non–NSTE-ACS indications, without an identifiable acute culprit lesion, or adjudicated as having type 2 myocardial infarction, 721 patients with NSTE-ACS and culprit-vessel characterization were retained. A further 233 patients were excluded for uncollected or incomplete serial troponin sampling or missing catheterization data, leaving 488 patients. Among patients who met the >90% stenosis criterion, five with no recorded TIMI flow grade were retained because the catheterization report documented 100% occlusion of the culprit vessel, and seven were excluded because they had neither a recorded TIMI flow grade nor documented 100% occlusion, leaving the angiographic OMI status unverifiable. The final analytic cohort comprised 481 patients (Figure 1).

**Figure 1:**
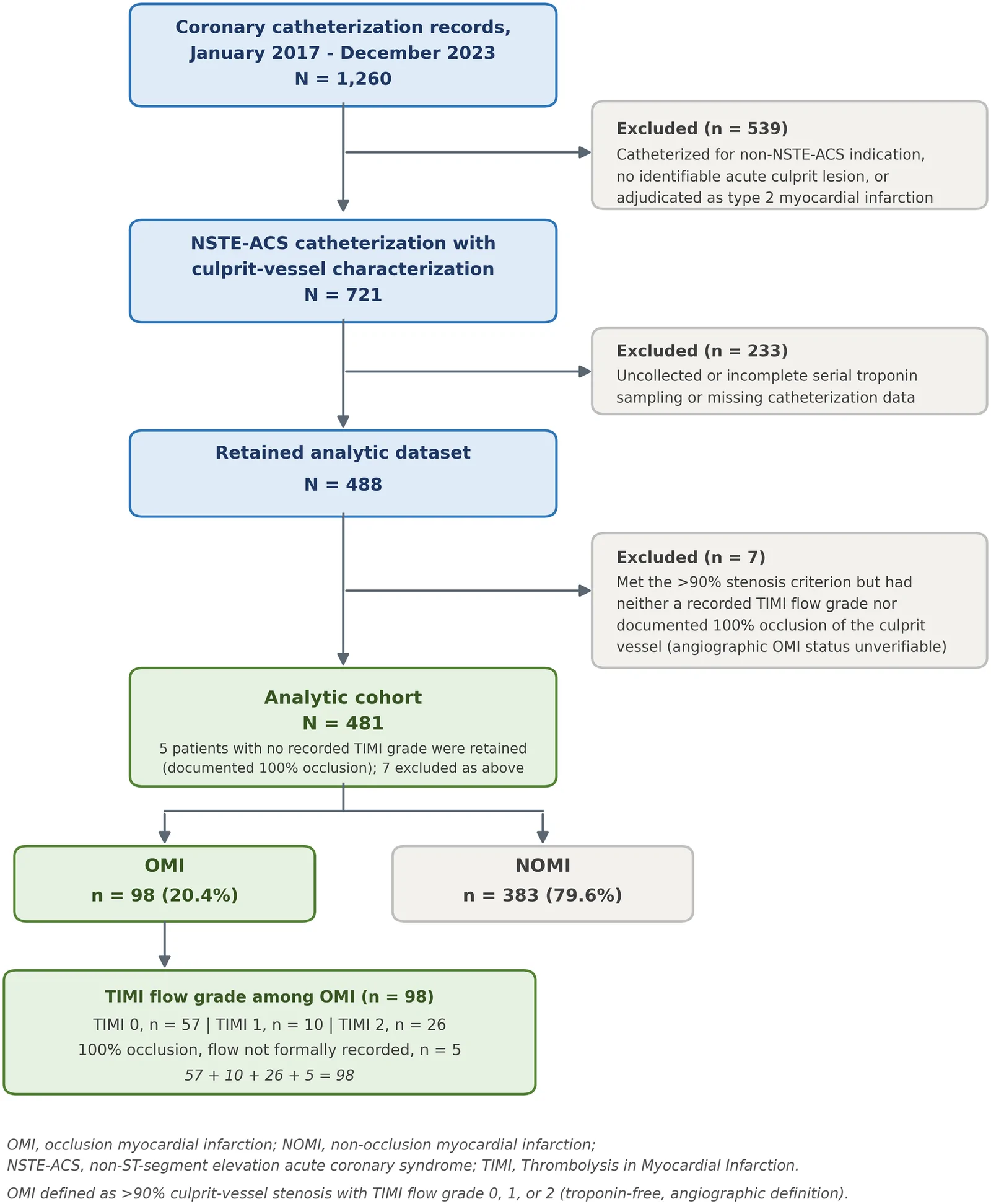
Patient flow diagram. Of 1,260 coronary catheterization records from January 2017 to December 2023, 721 represented non–ST-segment elevation acute coronary syndrome with culprit-vessel characterization after excluding 539 catheterized for a non–NSTE-ACS indication, without an identifiable acute culprit lesion, or adjudicated as type 2 myocardial infarction. A further 233 were excluded for uncollected or incomplete serial troponin sampling or missing catheterization data, leaving 488 patients in the retained analytic dataset. Seven patients who met the >90% stenosis criterion but had neither a recorded TIMI flow grade nor documented 100% occlusion of the culprit vessel were excluded as unverifiable, yielding a final analytic cohort of 481 patients [98 with occlusion myocardial infarction (20.4%) and 383 with non-occlusion myocardial infarction (79.6%)]. Five patients with no recorded TIMI grade but documented 100% occlusion were retained. Among the 98 patients with occlusion myocardial infarction, the TIMI flow grade was 0 in 57, 1 in 10, and 2 in 26, with flow not formally recorded in 5 who had documented 100% occlusion. Occlusion myocardial infarction was defined as >90% culprit-vessel stenosis with TIMI flow grade 0, 1, or 2, a troponin-free angiographic definition. OMI, occlusion myocardial infarction; NOMI, non-occlusion myocardial infarction; NSTE-ACS, non–ST-segment elevation acute coronary syndrome; TIMI, thrombolysis in myocardial infarction.

Among these patients, 98 (20.4%) met the angiographic definition of OMI and 383 (79.6%) were classified as having NOMI. TIMI flow grade distribution among the 98 patients with OMI is given as follows: TIMI 0 in 57 patients (58.2%), TIMI 1 in 10 (10.2%), TIMI 2 in 26 (26.5%), and TIMI flow not formally recorded but with 100% occlusion documented in 5 (5.1%).

### Baseline characteristics

Baseline demographic and clinical characteristics of the OMI and NOMI groups are presented in Table 1. Patients with OMI were younger than those with NOMI (median age, 65.5 vs. 70.0 years; *p* = 0.025) and had a lower prevalence of several chronic comorbidities, including heart failure (15.3% vs. 29.5%; *p* = 0.005), chronic lung disease (9.2% vs. 18.5%; *p* = 0.026), peripheral vascular disease (10.2% vs. 19.3%; *p* = 0.034), and prior coronary artery bypass grafting (5.1% vs. 12.5%; *p* = 0.036). Rates of hypertension, hyperlipidemia, diabetes mellitus, renal dysfunction, prior myocardial infarction, and prior PCI did not differ significantly between these groups.

**Table 1:** Baseline characteristics by OMI status.

Characteristic	OMI (*n* = 98)	NOMI (*n* = 383)	*p*-value
Age (years), median (IQR)	65.5 (57.2–75.8)	70.0 (59.0–78.5)	**0.025**
Female sex, *n* (%)	35/98 (35.7%)	128/383 (33.4%)	0.669
Race recorded as Caucasian, *n* (%)	86/98 (87.8%)	324/383 (84.6%)	0.431
Hypertension, *n* (%)	77/98 (78.6%)	329/383 (85.9%)	0.074
Hyperlipidemia, *n* (%)	74/98 (75.5%)	316/382 (82.7%)	0.103
Diabetes mellitus, *n* (%)	35/98 (35.7%)	174/383 (45.4%)	0.083
Renal dysfunction, *n* (%)	11/98 (11.2%)	58/383 (15.1%)	0.323
Peripheral vascular disease, *n* (%)	10/98 (10.2%)	74/383 (19.3%)	**0.034**
Chronic lung disease, *n* (%)	9/98 (9.2%)	71/383 (18.5%)	**0.026**
Heart failure, *n* (%)	15/98 (15.3%)	113/383 (29.5%)	**0.005**
Prior myocardial infarction, *n* (%)	25/98 (25.5%)	116/383 (30.3%)	0.354
Prior CABG, *n* (%)	5/98 (5.1%)	48/383 (12.5%)	**0.036**
Prior PCI, *n* (%)	30/98 (30.6%)	136/383 (35.5%)	0.363
Current/recent smoker, *n* (%)	26/97 (26.8%)	78/377 (20.7%)	0.194
Chest pain at presentation, *n* (%)	91/98 (92.9%)	317/383 (82.8%)	**0.013**
Active chest pain, *n*/*N* (%)	26/80 (32.5%)	92/342 (26.9%)	0.315
Dyspnea, *n* (%)	33/98 (33.7%)	146/383 (38.1%)	0.416
Nausea/vomiting, *n* (%)	12/98 (12.2%)	34/381 (8.9%)	0.320
Catheterization >24 h, *n* (%)	31/98 (31.6%)	186/383 (48.6%)	**0.003**
Cancer (history or active), *n* (%)	21/98 (21.4%)	68/382 (17.8%)	0.410
Reduced LV systolic function, *n*/*N* (%)	45/65 (69.2%)	128/223 (57.4%)	0.087
Multivessel disease, *n*/*N* (%)	35/94 (37.2%)	168/376 (44.7%)	0.192

CABG, coronary artery bypass grafting; LV, left ventricular; PCI, percutaneous coronary intervention; TIMI, thrombolysis in myocardial infarction.

OMI is defined as >90% culprit-vessel stenosis with TIMI flow grade 0, 1, or 2 (troponin-free, angiographic definition). Continuous variables are expressed as median (IQR) and compared using Mann–Whitney’s *U*-test; categorical variables are expressed as *n* (%) or *n*/*N* (%), where *N* is the number with the variable recorded, and compared using Pearson’s *χ*^2^ test (uncorrected). Active chest pain was recorded as a separate binary variable during abstraction and was available for 80 patients with OMI and 342 patients with NOMI; the precise operational wording used on the original abstraction form was not retained. Similarly, renal dysfunction, heart failure, and chronic lung disease were recorded as binary variables during the original standardized abstraction; the granular operational definitions used on the original form were not retained. Race categories reflect the terminology recorded in the retained analytic dataset; the method of ascertainment (self-reported vs. administrative coding) was not recorded. *p*-values <0.05 are shown in bold.

Chest pain at presentation was more common in the OMI group (92.9% vs. 82.8%; *p* = 0.013). Patients with OMI were also more likely to undergo catheterization within 24 h of presentation (68.4% vs. 51.4%; *p* = 0.003). There were no significant differences between groups in the prevalence of active chest pain, dyspnea, nausea or vomiting, smoking status, or cancer history.

### Angiographic findings and culprit vessel distribution

The circumflex/obtuse marginal territory was the most frequent culprit vessel among patients with OMI, accounting for 36 of 98 cases (36.7%), followed by the right coronary artery (31; 31.6%) and the left anterior descending artery (27; 27.6%). Three patients (3.1%) had ramus involvement, and one (1.0%) had left main disease (Table 2). Multivessel coronary artery disease was present in 37.2% of patients with OMI and 44.7% of patients with NOMI (*p* = 0.192). In a *post hoc* analysis restricted to 92 patients with OMI with a single >90% culprit lesion in the LAD, RCA, or OM/LCx territory, of whom 67 had a qualifying third troponin (24 OM/LCx, 25 RCA, and 18 LAD), the third troponin differed across territories, highest with circumflex and lowest with right coronary culprits (medians, 1.450, 0.831, and 0.447 ng/mL for OM/LCx, LAD, and RCA, respectively; Kruskal–Wallis; *p* = 0.032 across the 67 analyzable patients; Supplementary Table S1).

**Table 2:** Culprit lesion distribution among patients with occlusion myocardial infarction (*n* = 98).

Culprit vessel	*N*	% of OMI (*n* = 98)
OM/LCx	36	36.7
RCA	31	31.6
LAD	27	27.6
Ramus	3	3.1
Left main	1	1.0
Total	98	100.0

LCx, left circumflex artery; LAD, left anterior descending artery; OM, obtuse marginal branch; RCA, right coronary artery; TIMI, thrombolysis in myocardial infarction.

OMI is defined as >90% culprit-vessel stenosis with TIMI flow grade 0, 1, or 2 (troponin-free, angiographic definition). The culprit lesion was identified from the catheterization report as the vessel designated by the interventional cardiologist as responsible for the acute presentation.

### Serial troponin kinetics

Serial troponin values and trajectory metrics are presented in Table 3. The first troponin, drawn at presentation (0 h), did not differ significantly between OMI and NOMI groups (median, 0.107 vs. 0.080 ng/mL; *p* = 0.078). By the second draw (3 h), troponin levels were significantly higher in the OMI group (median, 0.523 vs. 0.203 ng/mL; *p* < 0.001), and this difference increased further at the third draw (6 h; median, 0.768 vs. 0.291 ng/mL; *p* < 0.001). A qualifying second draw was available in 85 of 98 patients with OMI (86.7%) vs. 360 of 383 patients with NOMI (94.0%; *p* = 0.015), and a qualifying third draw was available in 72 (73.5%) vs. 317 (82.8%; *p* = 0.037). Consistent with the sampling rule described in the Materials and methods section, later values were recorded only when a draw fell inside the specified window. The lower availability of later measurements was consistent with the earlier catheterization observed in the OMI group and with the prespecified sampling window protocol.

**Table 3:** Serial troponin kinetics by OMI status.

Troponin metric	OMI, median (IQR)	*n*	NOMI, median (IQR)	*n*	*p*-value
First troponin (0 h), ng/mL	0.107 (0.047–0.489)	98	0.080 (0.030–0.262)	381	0.078
Second troponin (3 h), ng/mL	0.523 (0.129–1.120)	85	0.203 (0.097–0.573)	360	**<0.001**
Third troponin (6 h), ng/mL	0.768 (0.256–1.855)	72	0.291 (0.110–0.864)	317	**<0.001**
Delta 0 → 3 h, ng/mL	0.141 (0.011–0.680)	85	0.067 (0.005–0.220)	360	**0.013**
Delta 0 → 6 h, ng/mL	0.357 (0.034–1.595)	72	0.111 (0.006–0.459)	317	**0.001**
Ratio (2nd/1st)	2.008 (1.091–9.383)	85	1.745 (1.049–4.975)	360	0.093
Ratio (3rd/1st)	3.892 (1.311–30.948)	72	2.303 (1.051–8.588)	317	**0.006**
Initially undetectable first troponin, *n*/*N* (%)	16/98 (16.3%)		51/381 (13.4%)		0.454
Undetectable-to-detectable conversion^a^, *n*/*N* (%)	15/16 (93.8%)		47/51 (92.2%)		1.000

Troponin I was measured with the Abbott ARCHITECT STAT Troponin-I assay (LOD, 0.01 ng/mL; 99th-percentile URL, 0.03 ng/mL). Results reported as undetectable were coded at the LOD (0.01 ng/mL), and results reported as “<0.030” (a detectable signal above the LOD but below the URL) were coded at 0.03 ng/mL. Serial samples were obtained at presentation (time 0) and within ±30 min of the protocol-specified 3- and 6-h time points; draws falling outside these windows were not abstracted, ensuring comparable intercollection intervals across patients (see the Materials and methods section). Delta = later value minus first value; ratio = later value ÷ first value. Values are expressed as median (IQR) and compared via Mann–Whitney’s *U*-test; *n* denotes patients with the relevant draw(s) available. *p*-values <0.05 in bold.

a Among patients with an initially undetectable first troponin (coded at the LOD; *n* = 16 OMI, *n* = 51 NOMI), the proportion converting to ≥0.03 ng/mL on the second or third draw, compared by Fisher’s exact test. Two NOMI patients lacked a first troponin value. The NOMI undetectable and conversion values were corrected during this revision after reverification against the locked analytic dataset (previously reported as 53/381, 13.9%, *p* = 0.523, and 49/53, 92.5%).

Troponin trajectory metrics demonstrated a steeper rise in patients with OMI. The delta troponin from 0 to 3 h was significantly greater in the OMI group (median, 0.141 vs. 0.067 ng/mL; *p* = 0.013), as was the delta from 0 to 6 h (median, 0.357 vs. 0.111 ng/mL; *p* = 0.001). The 3-h troponin ratio (second divided by the first value) was numerically higher in the OMI group but did not reach statistical significance (median, 2.008 vs. 1.745; *p* = 0.093). The 6-h ratio (third divided by the first value) was significantly higher in the OMI group (median, 3.892 vs. 2.303; *p* = 0.006).

The prevalence of an initially undetectable first troponin (coded at the LOD, 0.01 ng/mL) was similar between groups (16.3% in OMI vs. 13.4% in NOMI; *p* = 0.454). Nearly all patients with an undetectable first troponin concentration had a detectable troponin result on subsequent draws, with no difference in conversion rate between OMI and NOMI (93.8% vs. 92.2%; *p* = 1.000).

### Discriminative performance of troponin for OMI

Although 3- and 6-h troponin values were higher in patients with OMI, the overlap between groups was substantial, limiting their usefulness for classifying individual patients. The discriminative performance and operating characteristics of troponin metrics are presented in Table 4 and Supplementary Table S2. The prespecified primary predictor, the change in troponin concentration from 0 to 6 h (delta 0 → 6 h), showed only modest discrimination for OMI (AUC, 0.623; 95% CI, 0.544–0.703; DeLong *p* = 0.002 vs. the no-discrimination value of 0.50), with a confidence interval extending from near chance to well below a clinically useful level. Among the secondary metrics, the first troponin at presentation did not discriminate OMI from NOMI (AUC, 0.558; 95% CI, 0.491–0.624; *p* = 0.092). The second troponin (AUC, 0.626; 95% CI, 0.556–0.696; *p* = 0.0005), third troponin (AUC, 0.654; 95% CI, 0.580–0.727; *p* < 0.001), and delta 0 → 3 h (AUC, 0.586; 95% CI, 0.512–0.660; *p* = 0.023) were better than chance, but only just. The 3-h troponin ratio did not discriminate (AUC, 0.559; 95% CI, 0.487–0.630; *p* = 0.109); the 6-h ratio was statistically significant but weak (AUC, 0.603; 95% CI, 0.526–0.681; *p* = 0.009). No unadjusted troponin metric reached an AUC of 0.66. In paired comparison, the third troponin did not differ from the prespecified primary predictor (difference, +0.031; *p* = 0.328), exceeded the second troponin (*p* = 0.025), and exceeded the first (*p* = 0.002) (Supplementary Table S3, Panel B).

**Table 4:** Discrimination and operating characteristics of troponin metrics for occlusion myocardial infarction.

Panel A. Discrimination of troponin metrics for OMI (DeLong method)					
Predictor	*N*	Events	AUC	DeLong 95% CI	*p* vs. 0.50
Delta 0 → 6 h (primary)	389	72	0.623	0.544–0.703	0.002
First troponin (0 h)	479	98	0.558	0.491–0.624	0.092
Second troponin (3 h)	445	85	0.626	0.556–0.696	0.0005
Third troponin (6 h)	389	72	0.654	0.580–0.727	<0.001
Delta 0 → 3 h	445	85	0.586	0.512–0.660	0.023
Ratio (2nd/1st)	445	85	0.559	0.487–0.630	0.109
Ratio (3rd/1st)	389	72	0.603	0.526–0.681	0.009

Panel B. Cutoffs selected by the Youden index (hypothesis-generating)					
Predictor	Cutoff	Sens, %	Spec, %	PPV, %	NPV, %
Delta 0 → 6 h (primary)	0.917 ng/mL	37.5	87.1	39.7	86.0
First troponin (0 h)	0.286 ng/mL	39.8	76.4	30.2	83.1
Second troponin (3 h)	0.403 ng/mL	57.6	68.6	30.2	87.3
Third troponin (6 h)	0.379 ng/mL	69.4	57.7	27.2	89.3
Delta 0 → 3 h	0.233 ng/mL	44.7	75.3	29.9	85.2
Ratio (2nd/1st)	22.200	22.4	92.5	41.3	83.5
Ratio (3rd/1st)	24.333	33.3	87.7	38.1	85.3

CI, confidence interval; OMI, occlusion myocardial infarction.

AUC, area under the receiver operating characteristic curve, computed by trapezoidal integration; 95% confidence intervals and the test of each AUC against the no-discrimination value of 0.50 were computed by the DeLong method. *N* is the number of patients with the relevant troponin draw(s); events = OMI cases within that subset. The change in troponin from 0 to 6 h (delta 0 → 6 h) was the prespecified primary predictor; all other metrics were secondary. Delta = later value minus first value; ratio = later value ÷ first value. The variation in *N* across metrics reflects the troponin sampling windows described in the Materials and methods section.

NPV, negative predictive value; PPV, positive predictive value; Sens, sensitivity; Spec, specificity.

The Youden index–maximizing threshold (sensitivity + specificity − 1) is reported with sensitivity, specificity, positive predictive value, and negative predictive value, calculated from the observed 2 × 2 classification table for each predictor-specific analytic subset. Because each cutoff was both derived and evaluated in the same sample, these are apparent (in-sample) operating characteristics and are optimistically biased best-case estimates of out-of-sample performance; they are hypothesis-generating only, and external validation would be required before clinical application. 95% confidence intervals for these operating characteristics and positive and negative likelihood ratios are provided in Supplementary Table S2. Higher predictor values denote a greater likelihood of OMI.

At cutoffs selected based on the Youden index, the best-performing single metric was the third troponin (≥0.379 ng/mL), which achieved 69.4% sensitivity and 57.7% specificity, with a positive predictive value of 27.2% and a negative predictive value of 89.3%. Within the third troponin analytical subset (72 OMI cases among 389 patients; prevalence, 18.5%), a positive result at this cutoff corresponded to a post-test probability of 27.2%, while a negative result corresponded to a residual OMI probability of 10.7%. The prespecified primary predictor (delta 0 → 6 h) performed no better in clinical terms (sensitivity, 37.5%; specificity, 87.1%; positive predictive value, 39.7%; and negative predictive value, 86.0%). The highest positive predictive value was 41.3%, and fewer than 40% of OMI cases were identified by the first troponin, the only value available at initial triage, at its optimal threshold (sensitivity, 39.8%). No positive likelihood ratio exceeded 2.98, and no negative likelihood ratio fell below 0.53 (Supplementary Table S2). These operating characteristics, which are apparent in-sample estimates and therefore optimistically biased, indicate that troponin, whether as a single measurement or as a trajectory, does not usefully separate OMI from NOMI in this cohort (Table 4, Panel B; Figure 2). In a *post hoc* sensitivity analysis confined to the TIMI 0–1 subgroup of patients with OMI (*n* = 72) vs. the full NOMI group (*n* = 383), discrimination remained poor for every troponin metric, with no area under the curve exceeding 0.65 (third troponin AUC, 0.629; 95% CI, 0.542–0.716; delta, 0 → 6 h; AUC, 0.645; 95% CI, 0.561–0.730), and the first troponin again was non-discriminatory (AUC, 0.541; 95% CI, 0.465–0.617) (Supplementary Table S4). With the 26 patients with TIMI 2 instead reclassified as NOMI, AUCs ranged from 0.535 to 0.640 and no metric reached 0.65 (Supplementary Table S5). In the subgroup catheterized more than 24 h after presentation, in whom every serial sample was collected before catheterization, AUCs ranged from 0.484 to 0.605 and no metric differed significantly from chance (Supplementary Table S6).

**Figure 2:**
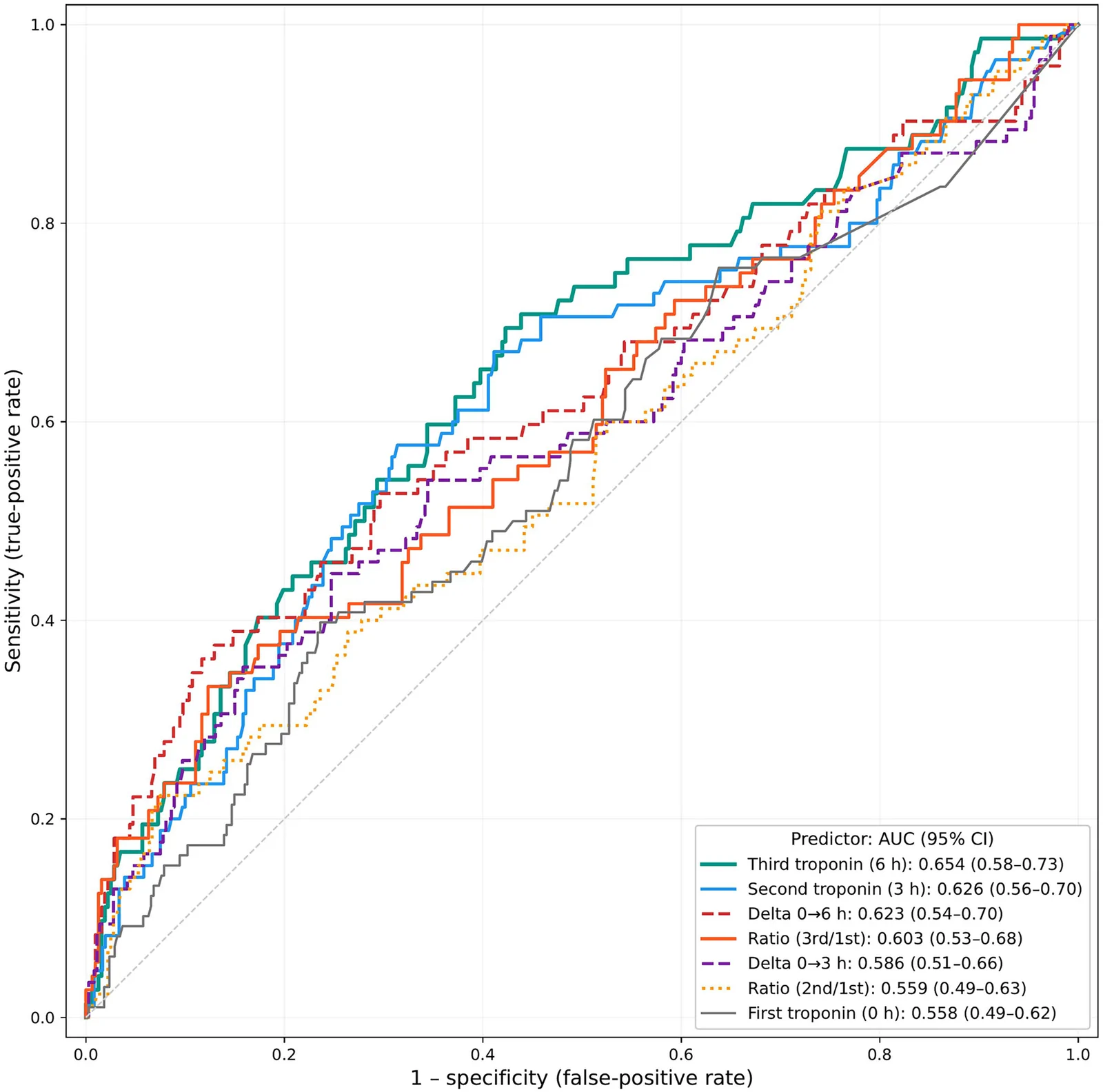
Receiver operating characteristic curves for troponin-based predictors of occlusion myocardial infarction. Seven predictors are shown: the first (0 h), second (3 h), and third (6 h) serial troponin measurements; the absolute changes from 0 to 3 h and from 0 to 6 h; and the third-to-first and second-to-first ratios. Areas under the curve with 95% confidence intervals were estimated using the DeLong method and are reported in the legend, ordered by discrimination. The 6-h troponin demonstrated the strongest discrimination (AUC, 0.654; 95% CI, 0.58–0.73), whereas the first troponin approximated the line of no discrimination (AUC, 0.558; 95% CI, 0.49–0.62). AUC, area under the curve; CI, confidence interval.

After adjustment for age and serum creatinine levels, the third troponin remained associated with OMI (odds ratio, 2.81 per 10-fold increase; 95% CI, 1.77–4.47; *p* < 0.001). The adjusted model reached an AUC of 0.702 (0.634–0.769) vs. 0.595 (0.525–0.666) for age and creatinine alone (Supplementary Table S3, Panel A). Stratified by renal dysfunction, the third-troponin AUC was 0.662 (0.583–0.740) in patients without renal dysfunction and 0.640 (0.433–0.847) in the small stratum with renal dysfunction (11 events), with the latter reported descriptively (Supplementary Table S3). Similarly, the prespecified primary predictor, modeled on its original scale, remained associated with OMI after adjustment [odds ratio, 1.56 per 1 ng/mL increase in delta 0 → 6 h; 95% CI, 1.27–1.91; *p* < 0.001; model AUC, 0.675 (0.602-0.748); *p* = 0.012 for the AUC increment over age and creatinine alone] and essentially unchanged after winsorization of extreme values (Supplementary Table S3, Panel A). The upper 95% confidence bounds were 0.703 for the prespecified primary predictor, 0.727 for the best individual metric, and 0.769 for the best exploratory adjusted model. Each adjusted model was developed and evaluated in its metric-specific available-case sample, and model AUCs are apparent in-sample estimates.

### Electrocardiographic and echocardiographic findings

Electrocardiographic findings are presented in Table 5. ECG data were available for 480 of 481 patients (99.8%). The most common finding in both groups was ST–T-wave changes (OMI, 60.2%; NOMI, 54.7%), followed by normal ECG (29.6% vs. 33.2%), LBBB (5.1% vs. 8.9%), and RBBB (5.1% vs. 3.1%). The overall distribution of ECG findings did not differ significantly between OMI and NOMI groups (*p* = 0.396).

**Table 5:** Electrocardiographic findings by OMI status.

ECG finding	OMI (*n* = 98)	NOMI (*n* = 382)	*p*-value
Normal, *n* (%)	29 (29.6%)	127 (33.2%)	0.396^a^
ST–T changes, *n* (%)	59 (60.2%)	209 (54.7%)	
LBBB, *n* (%)	5 (5.1%)	34 (8.9%)	
RBBB, *n* (%)	5 (5.1%)	12 (3.1%)	

LBBB, left bundle branch block; RBBB, right bundle branch block.

ECG findings were classified into four mutually exclusive categories. Adjudication was performed against the source ECG for any data-entry discrepancies; patients with a recorded ECG but no positive finding in any abnormal category were classified as normal. One NOMI patient had no ECG data available (NOMI *n* = 382).

a Pearson *χ*^2^ across all four categories.

Echocardiographic data were available for a subset of patients (288 of 481; 59.9%). Reduced left ventricular systolic function (EF <50%) was observed in 69.2% of patients with OMI and 57.4% of patients with NOMI with available data, a difference that did not reach statistical significance (*p* = 0.087; Table 1).

### Clinical outcomes

The in-hospital mortality rate was low and did not differ between the groups (3.1% in OMI vs. 2.6% in NOMI; *p* = 0.734; Table 6). Follow-up beyond the index hospitalization was incomplete: vital-status ascertainment reached at least 85% of the cohort only at the in-hospital and 2- and 5-year horizons. Ascertainment at the 30-day, 6-month, and 1-year horizons was lower because these depended on active clinical contact, whereas 2- and 5-year vital statuses were recovered through retrospective records review; the intermediate horizons are therefore not reported, and all mortality analyses are designated as exploratory. At 2 years, the all-cause mortality was 4.7% in the OMI group and 10.0% in the NOMI group (*p* = 0.140). At 5 years, the mortality rate was 10.5% vs. 20.0% (*p* = 0.041) under the carry-forward rule used in the original analysis for one death ascertained at 2 years, and it was 10.5% vs. 19.8% (*p* = 0.057) in an available-case analysis without that rule (Table 6). Nominal significance at 5 years was therefore not robust to the handling of one carry-forward death; the in-hospital and 2-year comparisons did not differ.

**Table 6:** All-cause mortality by OMI status (exploratory).

Outcome	OMI (*n* = 98)	NOMI (*n* = 383)	*p*-value
In-hospital death	3/98 (3.1%)	10/383 (2.6%)	0.734
2-year all-cause death	4/86 (4.7%)	33/331 (10.0%)	0.140
5-year all-cause death, carry-forward rule (primary)	9/86 (10.5%)	66/330 (20.0%)	**0.041**
5-year all-cause death, available-case (sensitivity)	9/86 (10.5%)	65/329 (19.8%)	0.057

Values are expressed as *n*/*N* (%), where *N* is the number of patients with vital status ascertained at that time point; *p* by Fisher’s exact test. Reporting is restricted to the three horizons with at least 85% cohort ascertainment—in-hospital (100%), 2-year (86.7%), and 5-year (86.3%); the 30-day, 6-month, and 1-year horizons depended on active clinical contact and did not reach this threshold, whereas 2- and 5-year vital statuses were recovered through retrospective records review. Two patients with NOMI had a 2-year vital status ascertained without a separate 5-year record: under the carry-forward rule used in the original analysis, one, recorded as deceased by 2 years, was carried forward as a death at 5 years (death being an absorbing state), and the other, alive at 2 years, was censored (NOMI 5-year *N* = 330). Direct 5-year vital-status ascertainment was 415/481 (86.3%); application of the carry-forward rule yielded an evaluable denominator of 416/481 (86.5%) for the primary 5-year comparison. The available-case row omits the carry-forward death (NOMI *N* = 329). Nominal significance at 5 years was not robust to this analytic choice. These analyses are exploratory, unadjusted, and not time-to-event estimates; the cohort was not powered for mortality. Given the younger age and lower comorbidity burden of the OMI group and the absence of adjustment, the difference most plausibly reflects baseline confounding and selection rather than a survival effect of coronary occlusion.

*p*-values <0.05 are shown in bold.

## Discussion

Our study showed three principal findings. First, no troponin metric, whether a single measurement or a serial trajectory, and including the prespecified primary predictor reliably discriminated OMI from NOMI. Second, approximately one in five analyzable patients met a purely angiographic, troponin-free definition of OMI. Third, the left circumflex artery was the most common culprit vessel among patients with OMI, and early troponin varied approximately threefold by culprit territory within the OMI group. We are not aware of a previous study that has assessed serial troponin against a purely angiographic OMI definition in a catheterized NSTE-ACS cohort.

The prevalence of OMI in our cohort falls at the lower end of the range reported in prior meta-analytic data, where estimates of occluded culprit prevalence range from 25% to 34% depending on the TIMI flow threshold and study population (5, 6). Our lower estimate is expected on definitional grounds, although selection in the analytic cohort may also contribute in either direction. The requirement of both >90% stenosis and reduced TIMI flow excludes slow flow without a high-grade proximal culprit, and patients who achieved spontaneous reperfusion to TIMI 3 before angiography are classified as NOMI, a real possibility in a cohort managed under a non-emergent NSTE-ACS strategy, as a large fraction of patients underwent catheterization more than 24 h after presentation. Patients with OMI form a sizeable minority of catheterized patients with NSTE-ACS whose acute pathology mirrors STEMI but whose management is not guided by emergent reperfusion protocols (10, 11).

Patients with OMI were younger, had fewer chronic comorbidities, and more often presented with chest pain (Table 1). This pattern, in which younger and less comorbid patients harbor worse acute pathology, has been consistently observed in prior studies of occluded culprit NSTEMI (5, 7). These differences should be regarded as descriptive. The greater frequency of catheterization within 24 h among patients with OMI may reflect presenting symptoms, clinician assessment, and referral and catheterization decisions rather than an intrinsic feature of occlusion, and restriction to an angiographically classifiable catheterized cohort with metric-specific available sampling introduces further selection. Whether risk scores that weigh age and comorbidity would assign appropriate urgency to such patients cannot be determined from these data (18, 19). In our cohort, the unadjusted 5-year mortality rate was numerically lower in the OMI group (Table 6), in contrast to prior reports in all-comer NSTEMI populations (5, 7). Several considerations may help explain the different direction of the observed association. The comparison is unadjusted, and marked baseline differences in age and comorbidity may substantially contribute to late survival. The cited studies estimated the association within all-comer populations, whereas this cohort is conditioned on referral to and survival until catheterization, and such selection may contribute to the different direction of the unadjusted association. Ascertainment was incomplete, no time-to-event modeling was performed, and nominal significance was not robust to the handling of a single carried-forward death. The finding is exploratory and should not be interpreted as evidence that occlusion is associated with better survival.

The circumflex artery was the most common OMI culprit in our cohort, followed by the right coronary and left anterior descending arteries (Table 2). Prior meta-analyses identified the circumflex artery as disproportionately represented among occluded culprit NSTEMI (5, 6), and Wei et al. reported an even stronger circumflex predominance when the definition was restricted to TIMI 0 flow (20). The posterolateral territory supplied by the circumflex is poorly represented by standard 12-lead ECG placement, and circumflex occlusion frequently produces ST depression rather than ST elevation, which would trigger emergent catheterization (4, 21). This finding is consistent with the anatomic premise of the OMI paradigm: some occlusions are missed because the involved territory is poorly represented on the standard 12-lead ECG. Within the OMI group, early troponin concentration also varied by territory, highest with circumflex and lowest with right coronary culprit lesions (Supplementary Table S1). Contrary to our expectation, circumflex occlusions were not associated with lower early troponin concentration. The comparison is *post hoc*, involves small numbers per territory, and is unadjusted; both the overall difference and its direction are hypothesis-generating. This within-group heterogeneity may contribute to the limited performance of any single troponin threshold because the range of values inside the OMI group overlaps the NOMI distribution wherever a cutoff is placed.

The troponin findings are best interpreted in three ways. First, the troponin concentration at presentation showed poor discriminative power. Because the symptom-onset time was unavailable, the extent to which this reflects heterogeneity in the interval between occlusion, symptom onset, and presentation cannot be determined (22). Second, serial measurements added information. The third troponin outperformed both earlier absolute measurements in a paired comparison, but the improvement remained modest and below the chosen interpretive benchmark. The prespecified primary predictor did not differ from the best single value. The failure of the delta to outperform the absolute value may partly reflect the subtraction of a presentation measurement with little discriminative information, which can add variability without adding signal. The clinically relevant role considered here is risk enrichment: whether troponin could help identify patients with NSTE-ACS who should be prioritized for expedited invasive evaluation, rather than being used as a standalone rule-in or rule-out test. The observed likelihood ratios produced only limited shifts in post-test probability and do not support standalone use; the negative predictive values should be interpreted in light of their dependence on OMI prevalence (Supplementary Table S2). Third, among the subgroup with an initially undetectable troponin, nearly all patients in both groups converted to a detectable result. Within this subgroup, the distinction between OMI and NOMI was defined by the magnitude of subsequent troponin release rather than by a detectable increase. Baro et al. reported a markedly higher discriminative performance for admission troponin in predicting total coronary occlusion (23); however, that study measured high-sensitivity troponin T (not the contemporary sensitivity troponin I assay used here), included patients with unstable angina, defined occlusion as TIMI 0 only, and adopted an optimal cutoff far exceeding the troponin concentrations available during the early decision-making window in our cohort. Differences in analyte, assay sensitivity, occlusion definition, and case mix limit direct comparison.

Biologically, troponin concentration reflects the extent and timing of myocardial injury, not whether the culprit artery is currently patent (22, 24). An occlusion in a modest territory may release less troponin than extensive injury from a non-occlusive culprit, and the territory analysis shows that the magnitude of injury varies widely even among confirmed occlusions. A biomarker of injury size cannot be expected to report vessel patency in the early hours of presentation. Comorbidities that raise baseline troponin levels, notably renal dysfunction, add further noise (16), and adjustment for age and creatinine did not alter the conclusion that discrimination remained modest. Closing this gap will likely require tools that assess occlusion more directly, rather than relying solely on biomarkers of myocardial injury (10, 11).

An important methodological strength of this study is that OMI was defined without using troponin. Prior studies, including the composite definition used by Meyers et al. (TIMI 0–2 flow, or TIMI 3 flow with high peak troponin), have incorporated troponin into both the predictor and the outcome (10), inflating performance estimates through incorporation bias (25). Our purely angiographic definition avoids directly incorporating troponin into the formal outcome definition. This approach also has an important limitation. Patients with transient occlusion and spontaneous reperfusion to TIMI 3 before angiography are classified as having NOMI. On average, occluded vessels produce greater myocardial injury and higher troponin levels than non-occlusive culprits, so this misclassification tends to place patients with comparatively high troponin levels into the NOMI comparator, narrowing the between-group troponin difference and making troponin appear less discriminating than it is. The magnitude of this effect is uncertain because early reperfusion can produce variable kinetics, a rapid washout spike, a blunted rise, or an intermediate trajectory depending on the timing of recanalization (22). The likely effect is making troponin appear less discriminating, not more. For that reason, the poor performance observed here is unlikely to be explained by an overly broad OMI definition. Separately, our angiographic criterion enriches the OMI group for severe epicardial occlusions, and sensitivity analyses that restrict the OMI group to TIMI 0–1 and reclassify TIMI 2 as NOMI both yield equally poor discrimination. The negative finding was robust to alternative handling of TIMI 2 flow, although these analyses cannot rule out other forms of angiographic outcome misclassification, including spontaneous reperfusion.

A negative study is informative only if it can be placed against a benchmark. For context, we considered an AUC of approximately 0.80 as a reasonable benchmark for a standalone triage biomarker in this setting. The upper 95% confidence bounds of the prespecified primary predictor, the best individual metric, and the exploratory adjusted models fell below this benchmark (see the Results section; Supplementary Table S3); each adjusted model was developed and evaluated in its metric-specific available-case sample. Within this study population, the data do not support discrimination approaching the level expected of a standalone triage biomarker.

The distribution of our four coarse ECG categories did not distinguish OMI from NOMI (Table 5). This analysis does not test expert OMI-specific ECG interpretation (8, 9). About three in ten angiographically confirmed occlusions (29.6%) had a presentation ECG classified as normal. This observation, more than the non-significant between-group comparison, motivates the call for better ECG-based identification. Normal here means no ST–T change or bundle branch block on coarse classification, not normal on expert review, which was not performed. In the meta-analysis conducted by de Alencar Neto et al., formal STEMI criteria had a pooled sensitivity of only 43.6% for detecting acute coronary occlusion, while OMI-specific ECG criteria substantially increased the sensitivity, with similar specificity (8). Emerging artificial intelligence models trained to detect OMI patterns have demonstrated high discriminative performance in international validation (26, 27), representing a promising avenue for closing the diagnostic gap highlighted by our data. Presentation ECG data were recorded for 480 patients in the original cohort. A future blinded dual-reader study using OMI-specific criteria and the source ECGs, with formal reliability assessment, would require renewed institutional approval and access to records; it is planned as a separate work.

## Limitations

This study has several limitations. First, it is a single-center retrospective analysis and requires multicenter prospective validation. Because the cohort comprises only patients who underwent cardiac catheterization, patients with NSTEMI who were managed medically, including those with contraindications, prohibitive comorbidity, or death before angiography, are not represented. The prevalence and troponin discrimination estimates therefore apply to the catheterized NSTE-ACS population and should not be extrapolated to the broader NSTEMI population.

Second, 233 of 721 potentially eligible NSTE-ACS catheterizations (32.3%) were excluded because of uncollected or incomplete serial troponin sampling or missing catheterization data. Among the remaining 488 patients, 7 additional patients were excluded because the angiographic OMI status could not be verified due to the absence of TIMI flow information without documented 100% culprit vessel occlusion. The study team has since left the study institution, and the IRB-approved protocol has expired, so no further abstraction of these records was possible. Individual-level data for the 233 previously excluded patients were not retained in the analytic dataset, so these patients could not be characterized. In addition, an included-vs.-excluded comparison could not be performed. Because no individual-level data are available for these patients, the direction and magnitude of the resulting selection bias cannot be determined. The prevalence estimate therefore applies to the final angiographically classifiable cohort, while diagnostic performance analyses are based on metric-specific available-case populations.

Third, the interval between each troponin draw and angiography or PCI was not recorded, so samples in patients catheterized early could not be classified as pre- or postprocedural. Postprocedural sampling could alter the magnitude and trajectory of later troponin values and bias diagnostic performance estimates in an uncertain direction. Importantly, discrimination remained similarly poor in the subgroup catheterized more than 24 h after presentation, in whom all evaluated serial samples necessarily preceded catheterization (Supplementary Table S6). Similarly, the time from symptom onset to the first troponin draw was not recorded. Because troponin release is a function of time since coronary occlusion (22), variability in the symptom-to-presentation interval adds noise to the presentation value, and its poor discrimination cannot be disentangled from this unmeasured confounder, as noted in the Materials and methods section.

Fourth, troponin concentration was measured using a contemporary sensitivity assay, and only early serial samples were captured. Peak troponin values were not abstracted; they generally occur after the early triage window and may be influenced by reperfusion, so they could not inform the preangiographic decision addressed in this study. Moreover, they can no longer be retrieved. Whether high-sensitivity kinetics would discriminate OMI more effectively remains untested, although the core conceptual limitation, that troponin indexes infarct size and timing rather than coronary patency, is assay-independent (22, 28). The trajectory analyses were further affected by non-random missingness. Second and third values were recorded only when a draw fell within the specified sampling window, so all delta and ratio metrics were undefined for patients without a qualifying later draw, and qualifying later draws were less often available in the OMI group (see the Results section). This shortfall is consistent with the earlier catheterization observed among patients with OMI and with the sampling protocol: patients catheterized before the later windows, possibly those with more severe occlusion, could not contribute to later draws and were absent from the trajectory comparisons and from the best-performing single metric. This selection, like the spontaneous-reperfusion misclassification inherent in the troponin-free outcome definition, could make troponin appear less discriminating, so the observed lack of discrimination may be conservative.

Fifth, the treatment received was not captured. Revascularizations performed, their modality, and their timing were not abstracted as dedicated variables, beyond a record of index-episode CABG in one patient and cardiothoracic surgery consultation in 32. The study therefore cannot directly evaluate the time to reperfusion or the consequences of treatment delay; it can describe only the time to catheterization. Similarly, the exploratory mortality comparisons cannot account for differences in treatment. The time to catheterization was anchored to presentation, whereas prior observational studies have more commonly anchored timing to the first positive troponin or symptom onset, so although our definition is guideline-aligned, the timing comparison is not directly comparable to those estimates.

Sixth, both the culprit designation and the TIMI flow grade derive from the report of the treating interventionalist, who was not blinded to clinical data, including available troponin results. No independent core laboratory adjudication or interobserver reliability assessment was performed, and neither is currently possible. TIMI flow grading is subjective and categorical (29), creating particular classification concerns near the TIMI 1–2 and 2–3 boundaries relevant to the outcome. Two sensitivity analyses bracket this uncertainty: restriction of the OMI group to TIMI 0–1 and reclassification of patients in TIMI 2 as NOMI, and the negative finding held in both (Supplementary Tables S4, S5), although neither is a substitute for blinded adjudication. Both analyses were *post hoc* rather than prespecified and should be interpreted accordingly.

Finally, the ECG analysis used broad categorical classifications rather than expert OMI-pattern interpretation and therefore does not address whether OMI-specific ECG reading would identify these patients; a blinded dual-reader study using OMI criteria is planned as separate work, as it requires renewed institutional approval and access to records. Follow-up was incomplete, and the mortality analyses are exploratory and unadjusted. Because the cohort was managed within a conventional STEMI/NSTEMI framework, the study cannot determine whether OMI-aware triage would improve outcomes.

## Conclusion

Among analyzable patients with NSTE-ACS undergoing catheterization, approximately one in five met the study-defined angiographic criteria for OMI. These patients were younger, less comorbid, and more likely to present with chest pain; these differences are descriptive. Early serial troponin kinetics showed only modest discrimination for OMI, with upper confidence bounds below levels expected of a standalone triage biomarker, and the circumflex artery was the most common culprit territory. These findings help explain why OMI remains difficult to recognize within the NSTEMI label. These findings support continued evaluation of complementary early identification strategies, including expert ECG interpretation, AI-assisted ECG tools, and point-of-care echocardiography.

## Data Availability

The datasets presented in this article are not readily available because the original institutional approval has expired and the data were collected under a single-site protocol. Requests to access the datasets should be directed to the corresponding author.
