# Supplementary material for: Angiographic prevalence of occlusion myocardial infarction and the limited value of serial troponin in patients with non–ST-segment elevation acute coronary syndrome
Source: Front Cardiovasc Med. 2026 Oct 5;13:1903711. doi: 10.3389/fcvm.2026.1903711 (PMC13677479; doi:10.3389/fcvm.2026.1903711)
Supplement: Supplementary file 1 [file Datasheet1.docx]

**Supplementary Table S1. Early troponin by major culprit territory within the OMI group, restricted to patients with a single >90% culprit lesion in the LAD, RCA, or LCx/OM territory (n = 92 of 98).**

| **Culprit territory** | **OMI, n** | **Third troponin (6 h), ng/mL, median (IQR)** | **n with draw** | **Delta 0→6 h, ng/mL, median (IQR)** | **n with draws** |
| --- | --- | --- | --- | --- | --- |
| LCx / OM | 36 | 1.450 (0.493–2.515) | 24 | 1.305 (0.144–2.500) | 24 |
| RCA | 30 | 0.447 (0.108–0.751) | 25 | 0.080 (0.007–0.366) | 25 |
| LAD | 26 | 0.831 (0.338–3.045) | 18 | 0.384 (0.031–2.816) | 18 |
| Kruskal–Wallis p (3 territories) |  | 0.032 |  | 0.012 |  |

Of 98 OMI patients, 95 had exactly one vessel with >90% stenosis; excluding two ramus and one left main culprits left 92 in the three major territories. The three patients with more than one >90% vessel are also omitted; two of these had major-territory culprit designations (one RCA, one LAD) and account for the difference between the 94 major-territory culprits in Table 2 and the 92 patients here, and the third had a ramus culprit. Of the 92, 67 had a qualifying third troponin (24 LCx/OM, 25 RCA, 18 LAD) and constitute the Kruskal–Wallis comparison; the same 67 contribute the delta values. Third-draw availability differs across territories (24/36 LCx/OM, 25/30 RCA, 18/26 LAD), consistent with differential catheterization timing, and territory medians should be read with this missingness in mind. Territory-specific AUCs are not reported because event counts per territory are insufficient for stable estimation. IQR, interquartile range; LAD, left anterior descending artery; LCx/OM, left circumflex/obtuse marginal; RCA, right coronary artery.

**Supplementary Table S2. Operating characteristics of troponin metrics at cut-offs selected by the Youden index, with 95% confidence intervals and likelihood ratios.**

| **Predictor** | **Cut-off** | **Sens, % (95% CI)** | **Spec, % (95% CI)** | **PPV, % (95% CI)** | **NPV, % (95% CI)** | **LR+ (95% CI)** | **LR− (95% CI)** |
| --- | --- | --- | --- | --- | --- | --- | --- |
| Delta 0→6 h (primary) | 0.917 ng/mL | 37.5 (27.2–49.0) | 87.1 (82.9–90.3) | 39.7 (28.9–51.6) | 86.0 (81.8–89.4) | 2.90 (1.92–4.38) | 0.72 (0.60–0.86) |
| First troponin (0 h) | 0.286 ng/mL | 39.8 (30.7–49.7) | 76.4 (71.9–80.4) | 30.2 (23.0–38.6) | 83.1 (78.9–86.7) | 1.68 (1.24–2.28) | 0.79 (0.66–0.93) |
| Second troponin (3 h) | 0.403 ng/mL | 57.6 (47.0–67.6) | 68.6 (63.6–73.2) | 30.2 (23.7–37.7) | 87.3 (82.9–90.7) | 1.84 (1.45–2.33) | 0.62 (0.48–0.80) |
| Third troponin (6 h) | 0.379 ng/mL | 69.4 (58.0–78.9) | 57.7 (52.2–63.0) | 27.2 (21.3–34.0) | 89.3 (84.3–92.8) | 1.64 (1.34–2.01) | 0.53 (0.37–0.76) |
| Delta 0→3 h | 0.233 ng/mL | 44.7 (34.6–55.3) | 75.3 (70.6–79.5) | 29.9 (22.6–38.4) | 85.2 (80.9–88.7) | 1.81 (1.34–2.43) | 0.73 (0.60–0.90) |
| Ratio (2nd/1st) | 22.200 | 22.4 (14.8–32.3) | 92.5 (89.3–94.8) | 41.3 (28.3–55.7) | 83.5 (79.5–86.8) | 2.98 (1.74–5.10) | 0.84 (0.75–0.94) |
| Ratio (3rd/1st) | 24.333 | 33.3 (23.5–44.8) | 87.7 (83.6–90.9) | 38.1 (27.1–50.4) | 85.3 (81.0–88.7) | 2.71 (1.75–4.20) | 0.76 (0.64–0.90) |

Confidence intervals for sensitivity, specificity, PPV, and NPV are Wilson score intervals; confidence intervals for likelihood ratios use the log method. Cut-offs, derived and evaluated in the same sample, are apparent in-sample estimates, optimistically biased, and hypothesis-generating only. The observed likelihood ratios produced only limited shifts in post-test probability and do not support use of any evaluated metric as a standalone rule-in or rule-out test. LR, likelihood ratio; NPV, negative predictive value; PPV, positive predictive value; Sens, sensitivity; Spec, specificity.

**Supplementary Table S3. Adjusted logistic models and paired comparisons of discrimination.**

**Panel A. Exploratory logistic models for OMI adjusted for age and serum creatinine.**

| **Model** | **N** | **Adjusted OR (95% CI)** | **p** | **Model AUC (95% CI)** | **Covariates-only AUC (95% CI)** | **Increment p** |
| --- | --- | --- | --- | --- | --- | --- |
| Third troponin (6 h) + age + creatinine | 389 | 2.81 per 10-fold (1.77–4.47) | <0.001 | 0.702 (0.634–0.769) | 0.595 (0.525–0.666) | 0.006 |
| Second troponin (3 h) + age + creatinine | 445 | 2.26 per 10-fold (1.52–3.37) | <0.001 | 0.683 (0.619–0.746) | 0.600 (0.535–0.664) | 0.009 |
| Delta 0→6 h (primary, original scale) + age + creatinine | 389 | 1.56 per 1 ng/mL (1.27–1.91) | <0.001 | 0.675 (0.602–0.748) | 0.595 (0.525–0.666) | 0.012 |

The second and third absolute troponin concentrations were entered as log10-transformed values, with odds ratios expressed per 10-fold increase; the prespecified delta 0→6-hour predictor was modeled on its original scale, with its odds ratio expressed per 1-ng/mL increase. Model AUCs represent apparent in-sample discrimination and were not internally or externally validated. The delta metrics can be negative or zero, so a log-scale model is undefined for them. The primary-predictor estimate was materially unchanged after winsorization at the 1st and 99th percentiles (OR 1.67; model AUC 0.674). Renal dysfunction refers to the binary variable recorded during the original abstraction; its granular operational definition was not retained. Increment p is the paired DeLong comparison of the full model against the covariates-only model. Stratified by renal dysfunction, the third-troponin AUC was 0.662 (0.583–0.740) in patients without renal dysfunction (N = 329, 61 events) and 0.640 (0.433–0.847) in patients with renal dysfunction (N = 60, 11 events), the latter reported descriptively given the event count. AUC, area under the curve; CI, confidence interval; OR, odds ratio.

**Panel B. Paired DeLong comparisons between troponin metrics on shared samples.**

| **Comparison** | **Shared N** | **Events** | **AUC A** | **AUC B** | **Difference** | **p** |
| --- | --- | --- | --- | --- | --- | --- |
| Third troponin vs delta 0→6 h (primary) | 389 | 72 | 0.654 | 0.623 | +0.031 | 0.328 |
| Second troponin vs delta 0→3 h | 445 | 85 | 0.626 | 0.586 | +0.040 | 0.235 |
| Third vs second troponin | 389 | 72 | 0.654 | 0.606 | +0.048 | 0.025 |
| Third vs first troponin | 389 | 72 | 0.654 | 0.504 | +0.150 | 0.002 |
| Delta 0→6 h vs first troponin | 389 | 72 | 0.623 | 0.504 | +0.119 | 0.065 |

Each comparison is computed in the subset with both metrics available, so AUCs may differ from the full-sample values in Table 4 Panel A. The prespecified primary predictor did not differ significantly from the best-performing single value. The third troponin outperformed both earlier absolute measurements in paired comparisons, indicating that serial sampling adds information, although the gain plateaus below clinical usefulness. AUC, area under the curve.

**Supplementary Table S4. Sensitivity analysis: OMI group restricted to TIMI 0–1 flow (n = 72) versus the full NOMI group (n = 383).**

| **Predictor** | **N** | **Events** | **AUC** | **DeLong 95% CI** | **p vs 0.50** |
| --- | --- | --- | --- | --- | --- |
| Delta 0→6 h (primary) | 369 | 52 | 0.645 | 0.561–0.730 | <0.001 |
| First troponin (0 h) | 453 | 72 | 0.541 | 0.465–0.617 | 0.287 |
| Second troponin (3 h) | 422 | 62 | 0.592 | 0.509–0.675 | 0.030 |
| Third troponin (6 h) | 369 | 52 | 0.629 | 0.542–0.716 | 0.004 |
| Delta 0→3 h | 422 | 62 | 0.607 | 0.528–0.687 | 0.008 |
| Ratio (2nd/1st) | 422 | 62 | 0.582 | 0.505–0.659 | 0.036 |
| Ratio (3rd/1st) | 369 | 52 | 0.619 | 0.535–0.702 | 0.005 |

The OMI group was restricted to TIMI 0 to 1 (57 with TIMI 0, 10 with TIMI 1, and 5 with documented 100% occlusion and TIMI flow not formally recorded); the 26 patients with TIMI 2 flow were excluded from this analysis. AUC, area under the receiver operating characteristic curve; CI, confidence interval; TIMI, Thrombolysis in Myocardial Infarction.

**Supplementary Table S5. Sensitivity analysis: TIMI 2 patients reclassified as NOMI (OMI n = 72, NOMI n = 409).**

| **Predictor** | **N** | **Events** | **AUC** | **DeLong 95% CI** | **p vs 0.50** |
| --- | --- | --- | --- | --- | --- |
| Delta 0→6 h (primary) | 389 | 52 | 0.640 | 0.557–0.723 | 0.001 |
| First troponin (0 h) | 479 | 72 | 0.535 | 0.460–0.610 | 0.364 |
| Second troponin (3 h) | 445 | 62 | 0.579 | 0.497–0.661 | 0.060 |
| Third troponin (6 h) | 389 | 52 | 0.617 | 0.530–0.703 | 0.008 |
| Delta 0→3 h | 445 | 62 | 0.604 | 0.526–0.682 | 0.009 |
| Ratio (2nd/1st) | 445 | 62 | 0.582 | 0.506–0.657 | 0.033 |
| Ratio (3rd/1st) | 389 | 52 | 0.614 | 0.532–0.696 | 0.007 |

The 26 OMI patients with TIMI 2 flow were reassigned to the NOMI comparator rather than excluded, the more conservative treatment of the occlusion boundary. Together with the TIMI 0–1 restriction in Supplementary Table S4, the two analyses bracket the boundary uncertainty; no metric reached an AUC of 0.65 in either. AUC, area under the receiver operating characteristic curve; CI, confidence interval; TIMI, Thrombolysis in Myocardial Infarction.

**Supplementary Table S6. Sensitivity analysis restricted to patients catheterized more than 24 hours after presentation (31 OMI, 186 NOMI), in whom all serial samples preceded angiography.**

| **Predictor** | **N** | **Events** | **AUC** | **DeLong 95% CI** | **p vs 0.50** |
| --- | --- | --- | --- | --- | --- |
| Delta 0→6 h (primary) | 199 | 30 | 0.605 | 0.487–0.723 | 0.081 |
| First troponin (0 h) | 216 | 31 | 0.484 | 0.368–0.601 | 0.794 |
| Second troponin (3 h) | 213 | 30 | 0.533 | 0.420–0.646 | 0.569 |
| Third troponin (6 h) | 199 | 30 | 0.594 | 0.485–0.704 | 0.092 |
| Delta 0→3 h | 213 | 30 | 0.548 | 0.433–0.663 | 0.414 |
| Ratio (2nd/1st) | 213 | 30 | 0.562 | 0.447–0.677 | 0.291 |
| Ratio (3rd/1st) | 199 | 30 | 0.595 | 0.475–0.715 | 0.120 |

Because catheterization occurred beyond 24 hours from the first troponin draw, every 0, 3, and 6 hour sample in this subgroup necessarily preceded angiography, removing any possibility of post-procedural influence on the index test. Discrimination was no better than in the full cohort and no metric differed significantly from chance. Interpretation is qualified by the selection inherent in delayed catheterization, the longer window for spontaneous reperfusion before angiography, and the limited number of events. AUC, area under the receiver operating characteristic curve; CI, confidence interval.
